# Exploration of biomarkers to predict clinical improvement of atopic dermatitis in patients treated with dupilumab: A study protocol

**DOI:** 10.1097/MD.0000000000022043

**Published:** 2020-09-18

**Authors:** Takeshi Nakahara, Kenji Izuhara, Daisuke Onozuka, Satoshi Nunomura, Risa Tamagawa-Mineoka, Koji Masuda, Susumu Ichiyama, Hidehisa Saeki, Yudai Kabata, Riichiro Abe, Mamitaro Ohtsuki, Koji Kamiya, Tatsuro Okano, Tomomitsu Miyagaki, Yozo Ishiuji, Akihiko Asahina, Hiroshi Kawasaki, Keiji Tanese, Hiroshi Mitsui, Tatsuyoshi Kawamura, Takuya Takeichi, Masashi Akiyama, Emi Nishida, Akimichi Morita, Kyoko Tonomura, Yukinobu Nakagawa, Koji Sugawara, Chiharu Tateishi, Yoko Kataoka, Rai Fujimoto, Sakae Kaneko, Eishin Morita, Akio Tanaka, Michihiro Hide, Natsuko Aoki, Shigetoshi Sano, Haruna Matsuda-Hirose, Yutaka Hatano, Motoi Takenaka, Hiroyuki Murota, Norito Katoh, Masutaka Furue

**Affiliations:** a Department of Dermatology, Graduate School of Medical Sciences, Kyushu University, Higashi-ku, Fukuoka; b Division of Medical Biochemistry, Department of Biomolecular Sciences, Saga Medical School, Saga; c Department of Preventive Medicine and Epidemiology, National Cerebral and Cardiovascular Center Research Institute, Suita, Osaka; d Department of Dermatology, Graduate School of Medical Science, Kyoto Prefectural University of Medicine, Kyoto; e Department of Dermatology, Nippon Medical School, Bunkyo-ku, Tokyo; f Division of Dermatology, Niigata University Graduate School of Medical and Dental Sciences, Niigata; g Department of Dermatology, Jichi Medical University, Shimotsuke, Tochigi; h Department of Dermatology, St. Marianna University School of Medicine, Kawasaki, Kanagawa; i Department of Dermatology, The Jikei University School of Medicine, Minato-ku; j Department of Dermatology, School of Medicine, Keio University, Shinjuku-ku, Tokyo; k Department of Dermatology, Faculty of Medicine, University of Yamanashi, Shimokato, Chuo-shi, Yamanashi; l Department of Dermatology, Nagoya University Graduate School of Medicine, Showa-ku; m Department of Geriatric and Environmental Dermatology, Nagoya City University Graduate School of Medical Sciences, Nagoya; n Department of Dermatology, Course of Integrated Medicine, Graduate School of Medicine, Osaka University; o Department of Dermatology, Osaka City University Graduate School of Medicine, Abeno-ku; p Department of Dermatology, Osaka Habikino Medical Center, Habikino City, Osaka; q Department of Dermatology, Shimane University Faculty of Medicine, Matsue, Shimane; r Department of Dermatology, Graduate School of Biomedical and Health Sciences, Hiroshima University, Minami-ku, Hiroshima; s Department of Dermatology, Kochi Medical School, Okatoyo-cho, Nankoku-shi, Kochi; t Department of Dermatology, Faculty of Medicine, Oita University, Hasama-machi, Yufu, Oita; u Department of Dermatology, Graduate School of Biomedical Sciences, Nagasaki University, Nagasaki City, Nagasaki, Japan.

**Keywords:** atopic dermatitis, biomarker, chemokines, cytokines, dupilumab, efficacy, treatment

## Abstract

**Background:**

Atopic dermatitis (AD) is a common eczematous skin disorder that profoundly reduces the quality of life due to intractable pruritus. Excellent therapeutic success of the anti-interleukin 4 receptor-α antibody dupilumab in clinical trials and a real-world clinical context indicates the crucial roles of interleukin (IL)-4 and IL-13 in the pathogenesis of AD. Along with the clinical improvement in skin scores and pruritus, dupilumab significantly and progressively reduces and normalizes the upregulated expression of T helper type 2 signatures such as Chemokine (C-C motif) ligand (CCL)17, CCL18, CCL22, and CCL26 in the lesional skin of AD. However, no blood/serum biomarkers are known to predict good or poor outcome in patients with AD treated with dupilumab.

**Methods:**

Patients are at least 18 years of age and have moderate-to-severe AD with Eczema Area and Severity Index (EASI) ≥16, Investigator's Global Assessment ≥3, and body surface area ≥10%. We are going to enroll more than 130 subjects from 18 medical facilities. Clinical objective findings will be evaluated by EASI. Subjective symptoms will be assessed by Patient-Oriented Eczema Measure, Numerical Rating Scale for Pruritus (Pruritus-NRS), Skin Comfort-NRS, and Treatment Satisfaction-NRS. We will measure 18 blood/serum biomarkers including % eosinophils in blood cell count, lactate dehydrogenase, total IgE, soluble interleukin 2 receptor, CCL17, CCL18, CCL22, CCL26, CCL27, IL-13, IL-22, IL-24, IL-25, IL-31, IL-33, thymic stromal lymphopoietin, periostin, and squamous cell carcinoma antigen-2. The clinical evaluation and biomarker sampling will be performed at 0, 2, 4, 8, and 16 weeks of dupilumab treatment. We will also perform proteomic analysis (of roughly 300 proteins) of the patients’ sera obtained at 0 and 2 weeks of treatment. The primary endpoint is the association between “baseline levels of 18 biomarkers” and “% change from baseline of EASI at 16 weeks of dupilumab treatment.”

**Discussion:**

This is the first clinical trial to explore the biomarkers, including potential proteomic markers, most strongly associated with improvement in EASI in patients with moderate-to-severe AD treated with dupilumab for 16 weeks (B-PAD study). A limitation is that we will only enroll Japanese patients.

## 1 Introduction

Atopic dermatitis (AD) is a common eczematous skin disorder, the incidence in the first 5 years of childhood of which is 10% to 16.5%. It is generally considered to have increased in prevalence worldwide, at least from the 1980 s to the early 2000 s.^[1]^ Clinical features of AD include skin inflammation, barrier dysfunction, and chronic pruritus.^[2]^ Its course involves chronic relapse with intense pruritus, which reduces the quality of life and decreases treatment satisfaction among afflicted patients.^[3–5]^ Excellent therapeutic success of the anti-interleukin 4 receptor-α antibody dupilumab in clinical trials and in a real-world clinical context has indicated the crucial roles of T helper type 2 (Th2) cytokines, interleukin (IL)-4 and IL-13, in the pathogenesis of AD.^[6–8]^ Along with the clinical improvement in skin scores and pruritus, dupilumab significantly and progressively reduces and normalizes the elevated expression of Th2 signatures such as Chemokine (C-C motif) ligand (CCL)17, CCL18, CCL22, and CCL26 in the lesional skin of AD.^[9,10]^ Other lesional and blood markers including eosinophils,^[11–13]^ lactate dehydrogenase (LDH),^[14]^ total immunoglobulin E (IgE),^[15]^ soluble IL-2 receptor,^[16]^ CCL27,^[14]^ IL-13,^[17]^ IL-22,^[9]^ IL-24,^[18,19]^ IL-25,^[20]^ IL-31,^[21,22]^ IL-33,^[23]^ thymic stromal lymphopoietin (TSLP),^[24]^ periostin,^[9,25]^ and squamous cell carcinoma antigen-2 (SCCA2)^[26,27]^ are elevated in AD and show substantial correlations with its disease activity.

It is now recognized that AD is not a single or monophenotypic disease, but is composed of heterogenous groups.^[11,28–30]^ In general, we have classified AD patients based on clinical features such as age (pediatric, young adult vs. elderly),^[30,31]^ clinical course (acute vs. chronic),^[32]^ IgE dependence (atopic vs. non-atopic),^[33,34]^ and ethnicity (Caucasian vs. non-Caucasian).^[35]^ In addition, recent approaches based on the molecular mechanisms have subdivided AD into different endoypes, for example, Th2 vs. Th2 + Th17,^[36–38]^ and clinical severity + Th2 / interferon-α/β.^[39]^ The phenotypic and endotypic differences in AD have led to a basis for stratifying patients. Stratifying patients by endotype may be particularly meaningful for the application of molecularly targeted drugs such as dupilumab. Although biomarkers representing the Th2 signature tend to decrease upon dupilumab treatment, the individual degrees of response of biomarkers as well as the rates of clinical improvement vary.^[40,41]^ In addition, it is not fully understood what kinds of biomarkers are responsible for a good/poor clinical outcome of dupilumab treatment.

The purpose of this study is to explore the biomarkers, including potential proteomic markers, that are most strongly associated with clinical improvement in patients with moderate-to-severe AD treated with dupilumab.

## 2 Methods/Design (Protocol version 1.0, registered on July 8th, 2019)

### 2.1 Study hypothesis/benefit

Certain biomarkers, including proteomic ones, may be associated with a good/poor clinical response to dupilumab. This information could be very useful for patients for whom the initiation of dupilumab therapy is being considered, given its high cost. Using meaningful stratification of patients, we can expect to increase efficacy of drugs and decrease the economic burden on patients. In addition, new Th2-related serum proteins may be highlighted by proteomic analysis as future target molecules in AD.

### 2.2 Study design

This is a multi-center, prospective, observational study in which samples/information will be obtained in Japan. This exploratory study will basically be carried out under real-world standard treatment guidelines. We are going to enroll more than 130 subjects from 19 medical facilities joining a consortium. The patients are to cease oral immunosuppressive drugs, oral steroids, or phototherapy at least 1 week before the start of injections of dupilumab. None of the patients is to have any previous experience of dupilumab treatment. They are to be at least 18 years of age, have moderate-to-severe AD with Eczema Area and Severity Index (EASI) ≥16, Investigator's Global Assessment (IGA) ≥3, and body surface area ≥10%, and be individuals for whom topical treatment of steroids provided inadequate control or was medically inadvisable, and had chronic AD for at least 3 years before the start of this study. The use of systemic steroids, systemic calcineurin inhibitors, and phototherapy is not allowed after the initiation of dupilumab.

The continued use of topical steroids, topical calcineurin inhibitors, topical moisturizers, and oral antihistamines used at baseline is allowed. Change of topical drugs to more potent ones is not allowed. The use of ocular, intranasal, or inhalant steroids and calcineurin inhibitors is allowed throughout the study, as is the use of anti-histamine drugs. Subjects are to receive subcutaneous injections of dupilumab (initial dose 600 mg, then 300 mg) biweekly for 16 weeks.

All investigators involved in this study shall carry out this study in accordance with the latest editions of the Declaration of Helsinki and “Ethical Guidelines for Medical and Health Research involving Human Subjects” of the Ministry of Health, Labour and Welfare, Japan. The study protocol has been approved by the Clinical Research Network Fukuoka Certified Review Board (CRB7180004). This study has been registered with the University Hospital Medical Information Network Clinical Trials Registry (UMIN000037307). The enrollment period is set to run from October 10, 2019. Last follow-ep date will be set on September 30, 2021.

### 2.3 Sample size estimates

The target number of 130 patients aimed to be enrolled was determined based on past experiences and feasibility. From previous phase 3 trials,^[7]^ since it is assumed that approximately 25% of enrolled patients with dupilumab treatment will discontinue the treatment, a plan was set to enroll more than 130 subjects and perform data analysis of at least 100 subjects.

### 2.4 Eligibility criteria

Inclusion criteria are as follows:

(1) chronic AD that has been present for ≥3 years at enrollment;
(2) moderate-to-severe patients with EASI score of ≥16, IGA score of ≥3, and body surface area ≥10% at enrollment (excluded if inflammation is limited to the head and neck region);
(3) no treatment history of dupilumab;
(4) patients in whom topical steroid treatment provides insufficient inhibition or is medically inadvisable;
(5) patients aged ≥18 years and ≤70 years at enrollment; and
(6) patients who are able to completely understand the study plan and to provide signed informed consent.

Exclusion criteria are as follows:

(1) patients treated with oral immunosuppressive drugs, oral steroid, or phototherapy within 4 weeks before dupilumab administration;
(2) female patients who are breastfeeding, pregnant, or have the possibility of being pregnant; and
(3) any other patients who are regarded as unsuitable for this study by the investigators.

Patient enrollment is performed by a central enrollment method. The investigators confirm that the study subjects meet all of the inclusion criteria and do not meet any of the exclusion criteria, and enter all of the necessary information for patient enrollment in the electronic data capture (EDC) system (Viedoc 4). Data monitoring including adverse events are periodically and independently performed by Clinical Research Support Center Kyushu (CReS Kyushu). Protocol kick-off meeting and amendment committee are also scheduled in the presence of CReS Kyushu.

### 2.5 Evaluation of clinical findings and biomarkers

**Figure 1:**
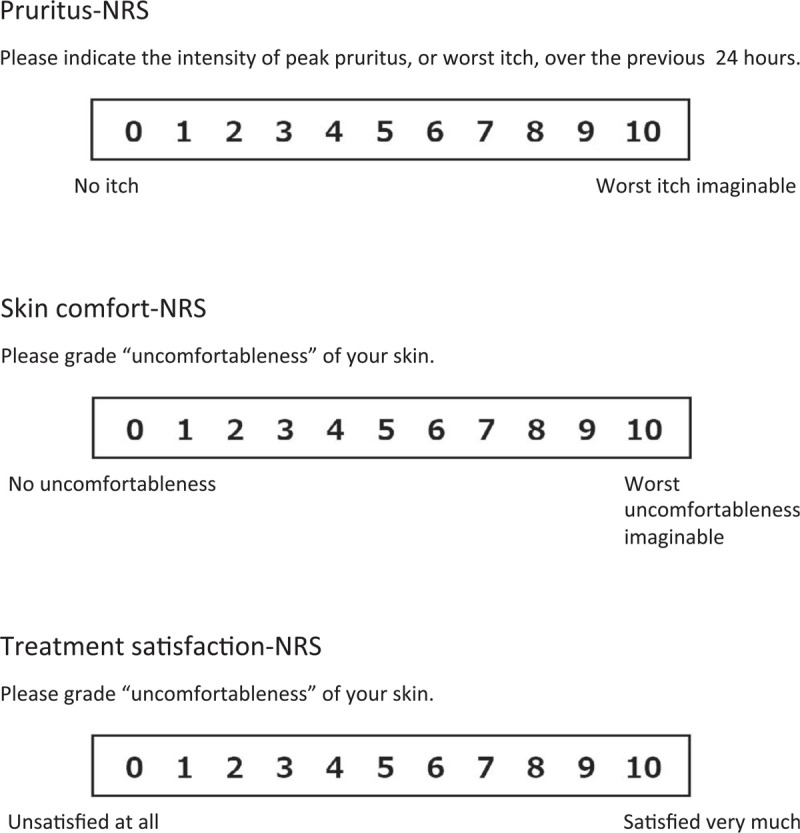
Pruritus-NRS, Skin comfort-NRS and Treatment satisfaction-NRS are used in this study. NRS = numerical rating scale.

**Figure 2:**
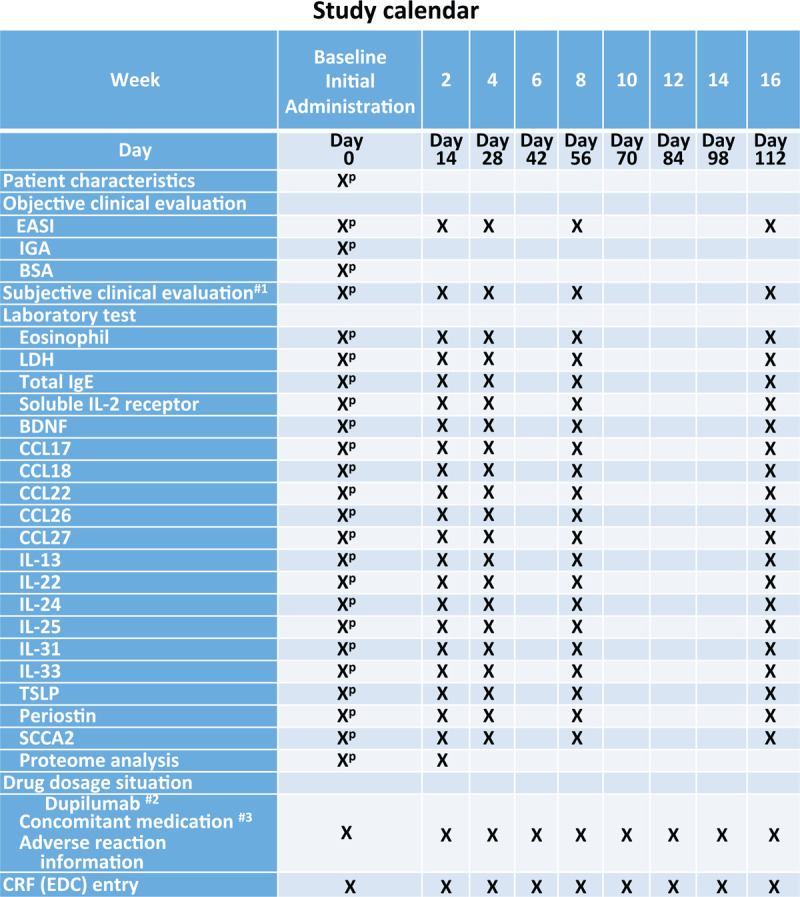
Study calendar is depicted. 1. Subjective clinical evaluation includes POEM, Pruritus-NRS, Uncomfortable skin-NRS, and Treatment satisfaction-NRS. 2. The administration of dupilumab shall be carried out after all assessments and tests are completed. A change of administration day is allowed within the range of +/- 1 week. #3. Use of ocular, intranasal, or inhalant steroids, calcineurin inhibitors, and antihistamines is allowed throughout the study. P = pre-treatment.

Clinical objective findings are evaluated by EASI.^[42–44]^ Subjective symptoms are assessed by Patient-Oriented Eczema Measure (POEM)^[44,45]^ and Numerical Rating Scale for Pruritus (Pruritus-NRS) (Fig. 1).^[46,47]^ Patients are also requested to complete Skin Comfort-NRS (0: no discomfort, 10: worst discomfort imaginable) and Treatment Satisfaction-NRS (0: not satisfied at all, 10: very satisfied) (Fig. 1). We measure 18 biomarkers including % eosinophils in blood cell count, LDH, total IgE, soluble interleukin 2 receptor, CCL17, CCL18, CCL22, CCL26, CCL27, IL-13, IL-22, IL-24, IL-25, IL-31, IL-33, TSLP, periostin, and SCCA2. The clinical evaluation and biomarker sampling are performed on the day that injections of dupilumab start and at 2, 4, 8, and 16 weeks (w) of dupilumab treatment (Fig. 2). We also perform proteomic analysis (of roughly 300 proteins) (Myriad RBM, Austin, TX) of the patients’ sera on the day that injections of dupilumab start and at 2w of treatment.

### 2.6 Primary and secondary endpoints

This is an exploratory clinical study to determine which biomarker is most strongly associated with clinical improvement. The primary endpoint is the association between “baseline levels of 18 biomarkers” and “% change from baseline of EASI at 16w of dupilumab treatment.” Secondary endpoints are

(1) the association between “baseline levels of potential proteomic markers” and “% change from baseline of EASI at 16w,”
(2) the association between “baseline levels of 18 biomarkers” and “% change from baseline of POEM at 16w,”
(3) the association between “baseline levels of potential proteomic markers” and “% change from baseline of POEM at 16w,”
(4) the association between “baseline levels of 18 biomarkers” and “% change from baseline of Pruritus-NRS at 16w,”
(5) the association between “baseline levels of potential proteomic markers” and “% change from baseline of Pruritus-NRS at 16w,”
(6) the association between “baseline levels of 18 biomarkers” and “% change from baseline of Skin Comfort-NRS at 16w,”
(7) the association between “baseline levels of potential proteomic markers” and “% change from baseline of Skin Comfort-NRS at 16w,”
(8) the association between “baseline levels of 18 biomarkers” and “% change from baseline of Treatment Satisfaction-NRS at 16w,” and
(9) the association between “baseline levels of potential proteomic markers” and “% change from baseline of Treatment Satisfaction-NRS at 16w.”

## 3 Statistical analysis

### 3.1 Relationship between biomarkers and clinical findings

To evaluate the primary and secondary endpoints, we will conduct 2 statistical procedures. First, we will check the distribution of the primary EASI and all secondary clinical findings (POEM, Pruritus-NRS, Skin Comfort-NRS, and Treatment Satisfaction-NRS). The primary EASI and all secondary subjective scores will be logarithmically transformed, and whether the data are normally distributed will be checked. “% change from baseline of EASI and all secondary clinical findings at 16w” will be referred to as the dependent variable, whereas “baseline level of each of the 18 biomarkers” will be referred to as the independent variable.

If we can assume that the data on the log-transformed endpoints at 16w are normally distributed, we will use an analysis of covariance model, adjusting for confounding factors. As potential confounding factors, sex, age, and medical and family history will be included in the model because these are known as important risk factors for AD.

If the data for the log-transformed endpoints at 16w do not fulfil the assumption of normality, we will use generalized linear models, adjusting for confounding factors such as sex, age, and medical and family history.

### 3.2 Relationship between potential proteomic markers and clinical findings

To evaluate the association between “baseline level of each potential proteomic marker” and “% change from baseline of primary (EASI) and all secondary clinical findings (POEM, Pruritus-NRS, Skin Comfort-NRS, and Treatment Satisfaction-NRS) at 16w,” we will also check the distribution of the primary EASI and all secondary clinical findings as mentioned above. “% change from baseline of the primary endpoint and all secondary clinical findings at 16w” will be referred to as the dependent variable, whereas “baseline level of each potential proteomic marker” will be referred to as the independent variable. Then, we will perform the same statistical analysis as described above.

### 3.3 Development of a score for evaluating disease activity in AD

Since measuring disease activity is an important component of AD management, biomarkers that capture the complex and heterogeneous biology of AD may have the potential to complement clinical disease activity assessment. We hypothesize that the measurement of multiple biomarkers and potential proteomic markers combined into a more limited score could quantitatively and objectively characterize AD activity and enhance AD activity assessment. Thus, after evaluating the associations of biomarkers and potential proteomic markers with primary and secondary endpoints, we will investigate the possibility of developing a score for evaluating disease activity in AD.

A score for disease activity in AD will be determined using the values of 18 biomarkers (% eosinophils, LDH, total IgE, soluble IL-2 receptor, CCL17, CCL22, CCL27, CCL18, CCL26, IL-13, IL-22, IL-24, IL-25, IL-31, IL-33, TSLP, periostin, and SCCA2) and potential proteomic markers (of roughly 300 molecules) during the 16w period of dupilumab treatment.

To evaluate the internal consistency of biomarkers and potential proteomic markers, Cronbach's α will be calculated. Mutual correlations of biomarkers and potential proteomic markers will be determined using correlation coefficients.

To explore potential groupings of the biomarkers and potential proteomic markers into a more limited number of score components, factorial analysis based on correlation coefficients will be performed. The selection of the number of score components will be based on the eigenvalues. To understand the meaning of the score components, promax rotation will be used. Finally, analysis of covariance or generalized linear models adjusting for confounding factors such as sex, age, and medical and family history will be used to evaluate the associations of combined scores with the primary endpoint and all secondary endpoints.

## 4 Discussion

The purpose of this study is to explore biomarkers that predict good and poor responders to dupilumab treatment in a real-world setting. As for the biomarkers, we will examine 18 candidates, all of which are known to be associated with disease activity of AD. For example, Guttman-Yassky et al. recently demonstrated that dupilumab treatment does significantly improve type 2 inflammatory signatures (IL-13, IL-31, CCL17, CCL18, and CCL26) in the blood and cutaneous tissues.^[9]^ Our previous studies also demonstrated that periostin and SCCA2 are downstream molecules of IL-4/IL-13 signaling and that these molecules are highly expressed in inflamed sites of AD patients.^[48–50]^ However, none of them has been analyzed as a predictor of response to dupilumab treatment. In addition, no stratification of AD patients to compare the efficacy of dupilumab was performed in 2 phase 3 trials of dupilumab for AD (SOLO1 and SOLO2).^[7]^ In these trials, the improvement as evaluated by IGA score as the primary outcome was 36% to 38%. In addition, the rate of achieving at least 75% improvement from baseline in EASI (EASI-75) as a secondary outcome was 44% to 51%.^[7]^ These results suggest that the efficacy of dupilumab varies among AD patients and that it is important to develop useful biomarkers to predict its efficacy, especially considering the economic burden on patients and the medical insurance cost of such treatment.

In asthma, recent studies have proposed several biomarkers to predict the efficacy of treatments. For example, asthma patients with baseline blood eosinophils of ≥300 cells per μL who are receiving high-dosage inhaled corticosteroids plus long-acting β2-agonists were reported to exhibit a longer exacerbation-free clinical course than those with placebo.^[51]^ Dupilumab was also shown to achieve substantial improvements in asthma patients with a baseline blood eosinophil count of at least 300 eosinophils per μL in terms of patient-reported outcomes such as morning and evening asthma symptom scores.^[52]^ In addition, the eosinophil count is a useful predictor of good treatment response in asthma patients treated with the anti-IL-5 antibody mepolizumab.^[53]^ The anti-IL-13 antibody lebrikizumab is also known to be efficacious for asthma treatment.^[54]^ Patients with high pretreatment levels of serum periostin have greater improvement in lung function upon lebrikizumab treatment than do patients with low periostin levels in asthma.^[54]^ Pretreatment serum levels of dipeptidyl peptidase-4 or periostin are also useful predictors of good therapeutic response in asthma patients administered the anti-IL-13 antibody tralokinumab.^[55]^

The present clinical trial will be the first to evaluate the pretreatment serum biomarkers that predict a good or poor outcome in patients with AD treated with dupilumab. Eighteen serum biomarkers that are known to reflect disease activity of AD are selected as potential candidates. We will also extend our study to seek new biomarkers using proteomic analysis. However, this study has a limitation that will only enroll Japanese patients. Recent reports suggest that patients of Asian origin with AD have a prominent IL-17 component.^[38]^ Therefore, there is a possibility that the findings of this study cannot be extrapolated to non-Asian AD. However, a biomarker assessment study is now ongoing in the European “BioDay” dupilumab treatment cohort.^[40]^ Although the primary endpoints differ, it will be possible to compare our results with those from “BioDay.”

## Author contributions

KI, DO, NK, TN, and MF conceived and designed and drafted the study protocol. All other authors approved the study protocol. TN and MF drafted the manuscript. All other authors critically reviewed the manuscript. All authors approved the final version of the manuscript.

## Abbreviations

Abbreviations: AD = atopic dermatitis, CCL = Chemokine (C-C motif) ligand, EASI = eczema area and severity index, IGA = Investigator's Global Assessment, IgE = immunoglobulin E, IL = interleukin, LDH = lactate dehydrogenase, NRS = numerical rating scale, POEM = patient-oriented eczema measure, SCCA2 = squamous cell carcinoma antigen-2, sIL-2R = soluble interleukin 2 receptor, Th2 = T helper type 2, TSLP = thymic stromal lymphopoietin.
